# Anterior segment optical coherence tomography in pupillary seclusion diagnosis and follow-up

**DOI:** 10.5935/0004-2749.20200113

**Published:** 2024-02-11

**Authors:** Jordi Izquierdo-Serra, Juan Pablo Figueroa-Vercellino, Marina Dotti-Boada, Néstor Ventura-Abreu, Marta Pazos

**Affiliations:** 1 Institut Clínic d’Oftalmología, Hospital Clínic de Barcelona, Universitat de Barcelona, Barcelona, Catalunya, Spain

Dear Editor,

Anterior segment optical coherence tomography (AS-OCT) has allowed the advancement toward a better understanding of the anatomy of the anterior segment, which is of capital importance in glaucoma care, allowing high-quality three-dimensional images of the anterior segment and dimension measurements of distances and areas^(1)^. These determinations aim for a better understanding of the dynamic changes in high-risk groups of patients, as in primary angle glaucoma disease^(2)^.

**Figure 1:**
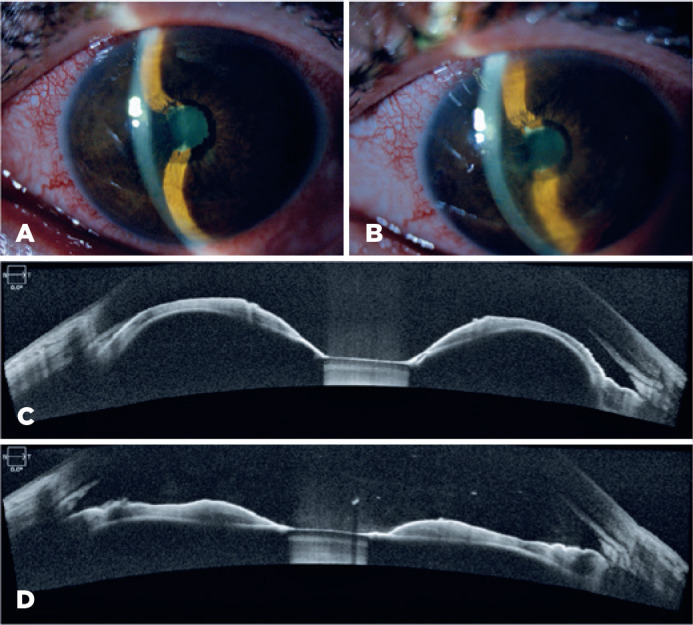
Photograph of the anterior segment showing an iris bombe due to a pupillary seclusion (A) and its resolution after laser peripheral iridotomy (LPI) (B). Wide angle-to-angle anterior segment optical coherence tomography (AS-OCT) demonstrating iris bombe with angle closure (C) and its resolution after LPI (D).

AS-OCT could also be of great utility in more straight forward, minimally invasive examinations in less frequent and clinically challenging conditions such as uveitic glaucomas. A 47-year-old man presented at the ophthalmology emergency department with eye pain and photophobia in his left eye (OS) in the previous week. He had consulted the department 2 weeks before for an episode of acute anterior uveitis in the same eye and was receiving treatment with topical corticoids and mydriatics. His visual acuity was 20/30. Slit-lamp examination revealed periciliary hyperemia, anterior chamber cells +2, 360^o^ posterior synechiae, and subsequent iris bombe appearance with peripheral iridocorneal contact (Figure 1A). Intraocular pressure (IOP) was 26 mmHg. Extensive synechiae precluded the fundus examination; therefore, ocular ultrasonography was performed, which ruled out a posterior pushing mechanism. AS-OCT detailed the angle closure caused by the anterior iris bowing secondary to seclusio pupillae (Figure 1C). After Nd:YAG laser peripheral iridotomy, AS-OCT revealed flattening of the iris with opening of the angle (Figure 1B and 1D). The IOP decreased to 18 mmHg and stabilized in low-teen values in the following visits. Iris bombe is an uncommon complication of uveitic glaucoma^(3)^ that presents with apposition of the iris to the lens, which prevents aqueous flow from the posterior to the anterior chamber. As a consequence, the pressure in the posterior chamber increases, causing anterior bowing of the peripheral iris and obstruction of the trabecular meshwork, which may result in an acute angle closure.

In most of these cases, iridotomy can restore aqueous humor outflow^(4)^. Although gonioscopy examination remains to be the gold standard technique for iridocorneal angle examination, recently, AS-OCT has proven to be a reliable high-resolution noninvasive imaging technique that can quantify not only the anterior chamber depth and volume^(4)^ but also the iris curvature and position, thus allowing angle closure diagnosis when irido-trabecular contact is visualized^(5)^. As illustrated in this case, when inflammation and poor collaboration hinder the gonioscopic examination, AS-OCT may be useful in performing a proper diagnosis and follow-up of this subgroup of angle closure.
